# Significant Associations of Achilles Tendon Thickness With Atherosclerotic Diseases and Atherosclerotic Risk Factors in Non-Familial Hypercholesterolemia Patients: A Cross-Sectional Study

**DOI:** 10.14740/jocmr6614

**Published:** 2026-09-26

**Authors:** Mariko Hakoshima, Hidekatsu Yanai, Tatsuki Onomura, Hirohumi Sugimoto, Hisayuki Katsuyama

**Affiliations:** a Department of Diabetes, Endocrinology and Metabolism, National Kohnodai Medical Center, Japan Institute for Health Security, Chiba, Japan; b Department of General Medicine, National Kohnodai Medical Center, Japan Institute for Health Security, Chiba, Japan; c Department of Diabetes, Endocrinology and Metabolism, National Center for Global Health and Medicine, Japan Institute for Health Security, Tokyo, Japan

**Keywords:** Achilles tendon thickness, Atherosclerotic risk factors, Diabetes, Cardiovascular disease, High-density lipoprotein

## Abstract

**Background:**

Achilles tendon thickness (ATT) is an important diagnostic tool for familial hypercholesterolemia (FH), a highly atherogenic genetic disorder. Although the available evidence remains limited, recent studies have reported associations between ATT and atherosclerotic diseases and their risk factors among non-FH patients. We investigated the relationships among ATT, carotid intima-media thickness (IMT), a marker of systemic atherosclerosis, and atherosclerotic risk factors, including diabetes, dyslipidemia, and atherosclerotic diseases, in non-FH patients.

**Methods:**

This cross-sectional survey was conducted at the National Kohnodai Medical Center, Japan. Patients who underwent ultrasonographic measurements of both ATT and carotid IMT were included. Clinical data were obtained using medical records and included demographic characteristics, anthropometric measurements, blood pressure, comorbidities, and biochemical parameters.

**Results:**

A total of 1,000 patients (512 males, 488 females) were enrolled. The mean age was 65 ± 14 years, the mean body mass index (BMI) was 24.6 ± 4.9 kg/m^2^, and the mean ATT was 4.6 ± 0.8 mm. ATT was positively correlated with maximal carotid IMT (r = 0.088, P = 0.005), body weight (r = 0.279, P < 0.001), and BMI (r = 0.183, P < 0.001), and inversely correlated with serum levels of high-density lipoprotein-cholesterol (HDL-C) (r = −0.182, P < 0.001). In the multiple regression model that included maximal carotid IMT, BMI, HDL-C, and low-density lipoprotein-cholesterol, the correlations of ATT with maximal carotid IMT, BMI, and HDL-C remained significant; however, the correlation between ATT and maximal carotid IMT was no longer significant in the model added with age. Patients with CAD and carotid artery stenosis had significantly greater ATT than those without such diseases. Similarly, ATT values were higher in patients with diabetes, and those with diabetes and CAD had the highest ATT values.

**Conclusion:**

ATT levels were significantly associated with BMI and dyslipidemia, and were significantly higher in non-FH patients with CAD, carotid artery stenosis, and diabetes than in non-FH patients without such diseases.

## Introduction

Achilles tendon thickness (ATT) is considered the most specific physical characteristic in familial hypercholesterolemia (FH), which is associated with premature atherosclerotic cardiovascular disease (ASCVD) [1]. This distinct presentation, resulting from extremely high serum low-density lipoprotein (LDL) levels and subsequent lipid accumulation, has been incorporated into diagnostic criteria worldwide. Traditionally, ATT has been evaluated by radiography as part of the diagnostic workup for FH [2–4]. In a study that assessed ATT in heterozygous FH patients (n = 1,273; 614 males, 659 females) using radiography [5], ATT changes were significantly associated with major adverse cardiovascular events (MACEs), suggesting that ATT assessment has diagnostic value and enables risk stratification of heterozygous FH patients [5].

Although the available evidence remains limited, recent studies have reported associations between ATT and atherosclerotic diseases and their risk factors among non-FH patients. The correlation between ATT, as measured by radiography, and the incidence of MACEs in patients with coronary artery disease (CAD) was investigated [6]. In this study, AT thickening was defined as having ATT of > 8.0 and > 7.5 mm in men and women, respectively. CAD patients with AT thickening had higher LDL-cholesterol (LDL-C) levels and incidence of MACEs than CAD patients without AT thickening, suggesting a significant contribution of high LDL-C levels to ATT and a significant association between ATT and MACEs in non-FH patients [6].

The association between ATT and coronary lesion severity was investigated in patients with early-onset acute coronary syndrome (ACS) without clinically diagnosed FH [7]. The severity of coronary lesions was positively correlated with the average ATT (r = 0.368, P = 0.0011) and the maximum ATT (r = 0.388, P = 0.0005), suggesting that ATT may predict CAD progression in non-FH patients [7].

Recently, ultrasonography has emerged as a non-invasive, radiation-free, and easily reproducible method for evaluating Achilles tendon morphology. Ultrasonographic cutoff values for the diagnosis of FH (5.8 mm in men and 5.5 mm in women) were determined [8]. In patients with heterozygous FH, MACE rates were higher in those with ATT detected by ultrasonography. However, classification based on ATT measured by radiography showed no difference in MACE rates between patients with and without AT thickening [9], suggesting that ultrasonography-determined ATT is superior to radiography-determined ATT in predicting MACEs.

A very recent study that assessed the relationship between ATT and CAD severity in patients with ACS without FH found that ATT, particularly ultrasonography-determined ATT, was significantly associated with CAD severity, suggesting that ultrasonography-determined ATT may serve as a practical, non-invasive marker for cardiovascular risk stratification [10].

Although growing evidence supports the usefulness of ultrasonographic ATT measurement in both FH and non-FH patients, the association of ATT with atherosclerotic diseases and coronary risk factors remains unclear.

We investigated the association between ATT and atherosclerotic or atherogenic diseases, including CAD, cerebrovascular disease, carotid artery stenosis, and diabetes. Furthermore, we examined the correlation between ATT, measured by ultrasonography, and carotid intima-media thickness (IMT), a marker of systemic atherosclerosis [11], as well as the correlation between ATT and metabolic parameters.

## Materials and Methods

### Study design and subjects

This retrospective observational study was conducted at the National Kohnodai Medical Center, Japan Institute for Health Security (JIHS), Chiba, Japan. We reviewed the electronic medical records of patients who underwent Achilles tendon ultrasonography in conjunction with carotid ultrasonography between November 1, 2018, and June 1, 2025. Demographic data (age, sex, body weight, and body mass index (BMI)), as well as clinical parameters (blood pressure, comorbidities, and laboratory results), were collected. Patients with a documented diagnosis of FH in electronic medical records were excluded. In addition, we re-reviewed the medical records of patients with markedly elevated LDL-C levels and/or increased ATT to identify possible FH cases; any patients subsequently confirmed to have FH were also excluded from the analysis. There were no inclusion criteria based on LDL-C levels; all patients who underwent both Achilles tendon and carotid ultrasonography during the study period were eligible, after excluding patients with FH. Diabetes was diagnosed based on either antidiabetic medication use or a hemoglobin A1c (HbA1c) level > 6.5%.

### Laboratory measurements

#### Anthropometric and blood pressure measurements

Body weight, body height, and blood pressure were measured according to the recommendations of the World Health Organization and the Japanese Society of Hypertension clinical guidelines.

#### Blood biochemical tests

HbA1c, total cholesterol (TC),triglyceride (TG), LDL-C, and high-density lipoprotein-cholesterol (HDL-C) were measured using enzymatic assays. Plasma glucose was measured using the hexokinase method. We calculated serum non-HDL-C levels by subtracting HDL-C from TC.

#### ATT measurement by ultrasonography

ATT was measured using ultrasonography, performed during routine carotid ultrasound examinations. Measurements were obtained at the level of the medial malleolus using a standardized longitudinal view, and the maximum tendon thickness on each side was recorded [8]. Participants were positioned either kneeling on the examination bed or prone. In the prone position, the limb was placed in either a neutral or dependent posture. The neutral position was defined as the foot perpendicular to the floor, with the ankle at 90° relative to the lower leg axis. A sufficient amount of ultrasound gel or a gel pad was applied, and the probe was positioned perpendicular to the long axis of the foot, maintaining a 90° angle between the skin and the probe surface. The probe was then slowly moved from caudal to cranial to visualize the Achilles tendon, and measurements were taken at the site of the greatest thickness.

#### Carotid IMT measurement

Carotid IMT was measured according to the Mannheim carotid IMT and plaque consensus (2004-2006-2011) [12]. Carotid IMT measurements were performed by one physician and five experienced medical sonographers in accordance with the Mannheim carotid IMT consensus. Using B-mode ultrasonography, the far wall of the common carotid artery was assessed approximately 1–2 cm proximal to the carotid bulb. Both carotid arteries were assessed, and mean values were calculated. Maximum IMT was manually determined at three measurement points. Carotid artery stenosis was defined as ≥ 30% stenosis of the carotid artery, corresponding to moderate (30–69%) or severe (70–99%) stenosis, according to the NASCET criteria [13].

### Statistical analysis

Statistical analyses were performed using IBM SPSS Statistics for Windows (Version 22.0; IBM Corp., Armonk, NY). Continuous variables were expressed as mean ± standard deviation (SD), and categorical variables were summarized as frequencies and percentages. Group comparisons, including the presence or absence of diabetes or cardiovascular disease (CVD), were analyzed by the Mann–Whitney U test. Correlations between ATT and clinical parameters were analyzed by Spearman’s rank correlation. Multiple regression analysis was performed on factors that showed significant correlations in the univariate analysis. A two-sided P value < 0.05 was considered statistically significant.

### Ethical statements

This study was conducted in accordance with the Declaration of Helsinki. The research protocol was reviewed by the JIHS Ethics Committee and approved under Approval Number JIHS-S-005091-00. As this study was a retrospective study using anonymized clinical data, informed consent from individual subjects was not required. Instead, study information was disclosed on the institutional website, and informed consent was obtained using an opt-out approach.

## Results

### Characteristics of subjects studied

A total of 1,000 patients (512 males, 488 females) were included in this study. Baseline demographic and clinical data are presented in Table 1. Among the study population, 8.8% had a history of CAD, 15.2% had cerebrovascular disease, and 11.4% had carotid artery stenosis. Diabetes was present in 64% of the patients. Pharmacotherapies for dyslipidemia, hypertension, and diabetes are shown in Table 2.

**Table 1:** Clinical Characteristics of the Studied Patients

Clinical characteristics	
Age (years)	64 ± 14
Gender (male/female)	512/488
Body height (cm)	161 ± 10
Body weight (kg)	64 ± 16
BMI (kg/m^2^)	24.6 ± 4.9
Systolic blood pressure (mm Hg)	131 ± 20
Diastolic blood pressure (mm Hg)	77 ± 13
Comorbidities	
Coronary artery disease (n, %)	88/998, 8.8
Cerebrovascular disease (n, %)	152/998, 15.2
Carotid artery stenosis (n, %)	114/1,000, 11.4
Diabetes (n, %)	643/997, 64.0

Values are expressed as mean ± SD. BMI: body mass index; SD: standard deviation.

**Table 2:** Pharmacotherapies for Dyslipidemia, Hypertension, and Diabetes

Pharmacotherapies	
Anti-hyperlipidemic drugs	
Statins	353 (37%)
Fibrates	55 (6%)
Omega-3 fatty acids	47 (5%)
Ezetimibe	64 (7%)
Probucol	1 (0.1%)
Anti-hypertensive drugs	
RAS inhibitors	277 (29%)
CCBs	302 (31%)
β-blockers	73 (8%)
α-blockers	14 (1%)
MRAs	28 (3%)
Diuretics	52 (5%)
Anti-diabetic drugs	
Biguanide	217 (23%)
DPP4 inhibitors	270 (28%)
SGLT2 inhibitors	192 (20%)
GLP-1RAs	59 (6%)
Thiazolidinediones	66 (7%)
α-GIs	48 (5%)
Insulin	115 (12%)

α-GIs: α-glucosidase inhibitors; CCBs: calcium channel blockers; DPP4: dipeptidyl peptidase 4; GLP-1RAs: glucagon-like peptide 1 receptor agonists; MRAs: mineralocorticoid receptor antagonists; RAS: renin-angiotensin system; SGLT2: sodium-glucose co-transporter 2.

### Ultrasonographic measurements and metabolic parameters

Ultrasonographic measurements of the carotid artery and Achilles tendon, along with biochemical data, are shown in Table 3. The mean maximal carotid IMT was 1.8 mm, which was greater than that reported in Japanese patients with non-cardioembolic stroke and hyperlipidemia in the J-STARS Echo Study (1.19 ± 0.32 mm) [14].

**Table 3:** Ultrasonographic Measurements of the Carotid Artery and Achilles Tendon and Biochemical Data

Ultrasonographic measurements	
Maximal IMT of carotid artery (mm)	1.8 ± 1.0
Achilles tendon thickness (mm)	4.6 ± 0.8
Biochemical data	
LDL-C (mg/dL)	115 ± 39
HDL-C (mg/dL)	54 ± 17
Triglyceride (mg/dL)	145 ± 90
Non-HDL-C	142 ± 43
Plasma glucose (mg/dL)	167 ± 92
HbA1c (%)	8.2 ± 2.7

Values are expressed as mean ± SD. HbA1c: hemoglobin A1c; HDL-C: high-density lipoprotein-cholesterol; IMT: intima-media thickness; LDL-C: low-density lipoprotein-cholesterol; SD: standard deviation.

The mean ATT was 4.6 mm, which was below the ultrasonographic cutoff values proposed for the diagnosis of FH (5.8 mm in men and 5.5 mm in women) [8]. Mean serum lipid levels were within the normal range. In contrast, mean plasma glucose and HbA1c values were elevated, reflecting the relatively high proportion of patients with diabetes and suboptimal glycemic control.

### Correlations between ATT, carotid IMT, and metabolic parameters

The correlations between ATT, carotid IMT, and metabolic parameters in all patients and patients with diabetes are shown in Tables 4 and 5, respectively. Maximal carotid IMT was significantly and positively correlated with ATT in both the overall cohort and the subgroup with diabetes. In addition, body weight and BMI were significantly and positively correlated with ATT levels in both groups. Serum HDL-C levels were inversely and significantly correlated with ATT levels in both groups. We did not find any significant association between ATT and other metabolic parameters.

**Table 4:** Correlations of Achilles Tendon Thickness With Carotid Artery IMT and Metabolic Parameters in All Patients (n = 1,000)

	Correlation coefficient	P values
Maximal carotid IMT	0.09	0.005
Age	−0.001	0.983
Body weight	0.28	< 0.001
BMI	0.18	< 0.001
Systolic blood pressure	0.05	0.131
Diastolic blood pressure	0.06	0.084
LDL-C	−0.05	0.206
HDL-C	−0.18	< 0.001
Triglyceride	0.06	0.066
Non-HDL-C	−0.04	0.239
Plasma glucose	0.04	0.240
HbA1c	0.01	0.766

Values are expressed as mean ± SD. BMI: body mass index; HbA1c: hemoglobin A1c; HDL-C: high-density lipoprotein-cholesterol; IMT: intima-media thickness; LDL-C: low-density lipoprotein-cholesterol; SD: standard deviation.

**Table 5:** Correlations of Achilles Tendon Thickness With Carotid Artery IMT and Metabolic Parameters in Patients With Diabetes (n = 643)

	Correlation coefficient	P values
Maximal carotid IMT	0.12	0.002
Age	0.003	0.939
Body weight	0.26	< 0.001
BMI	0.16	< 0.001
Systolic blood pressure	0.06	0.120
Diastolic blood pressure	0.07	0.095
LDL-C	0.01	0.868
HDL-C	−0.14	< 0.001
Triglyceride	0.08	0.832
Non-HDL-C	−0.04	0.282
Plasma glucose	0.02	0.651
HbA1c	−0.06	0.170

Values are expressed as mean ± SD. BMI: body mass index; HbA1c: hemoglobin A1c; HDL-C: high-density lipoprotein-cholesterol; IMT: intima-media thickness; LDL-C: low-density lipoprotein-cholesterol; SD: standard deviation.

### Multiple regression analysis with ATT as the dependent variable

We performed multiple regression analysis to examine the associations of ATT—treated as the dependent variable—with maximal carotid IMT, BMI, HDL-C, and LDL-C. Maximal carotid IMT was significantly and positively correlated with ATT levels (β = 0.111, P = 0.003). BMI was significantly and positively correlated with ATT levels (β = 0.174, P < 0.001). Serum HDL-C levels were inversely and significantly correlated with ATT levels (β = −0.127, P = 0.001). In the model that included age as an explanatory variable, the correlations of ATT with BMI and HDL-C remained significant; however, the significant correlation between ATT and maximal carotid IMT was no longer significant.

### ATT and atherosclerotic diseases

Comparisons of ATT between patients with and without CAD are shown in Figure 1. ATT was significantly greater in patients with CAD than in those without CAD across all patients, patients older than 65 years, female patients, and patients treated with statins.

**Figure 1:**
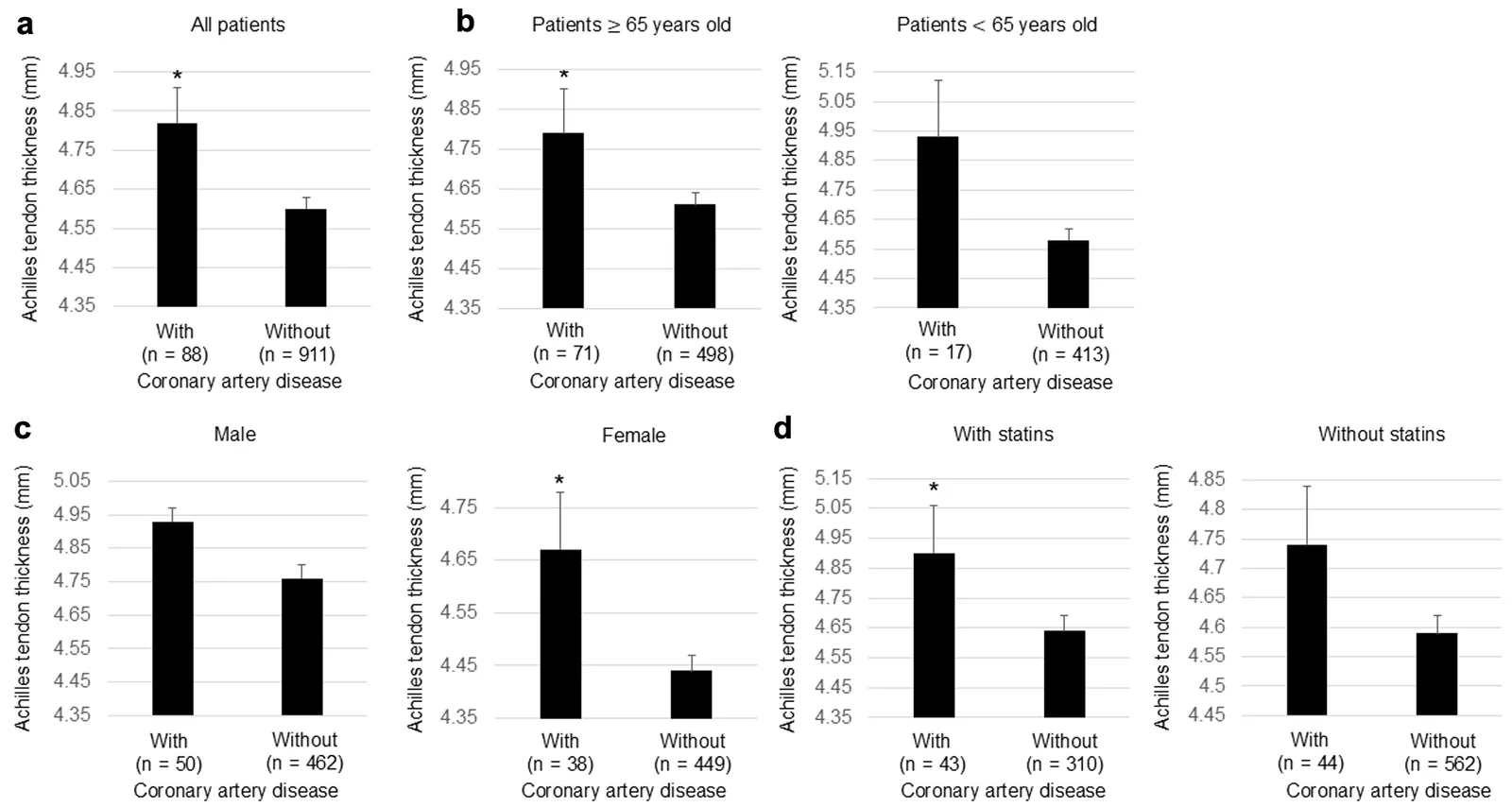
Achilles tendon thickness in patients with and without coronary artery disease in all patients (a), in patients ≥ 65 years old and patients < 65 years old (b), in male and female patients (c), and in patients treated with statins and patients untreated with statins (d). Error bars indicate mean ± SE. *P < 0.01 vs. patients without coronary artery disease. SE: standard error.

Comparisons of ATT between patients with and without cerebrovascular disease are shown in Figure 2. There was no significant difference in ATT between patients with and without cerebrovascular disease across all patients and all subgroups.

**Figure 2:**
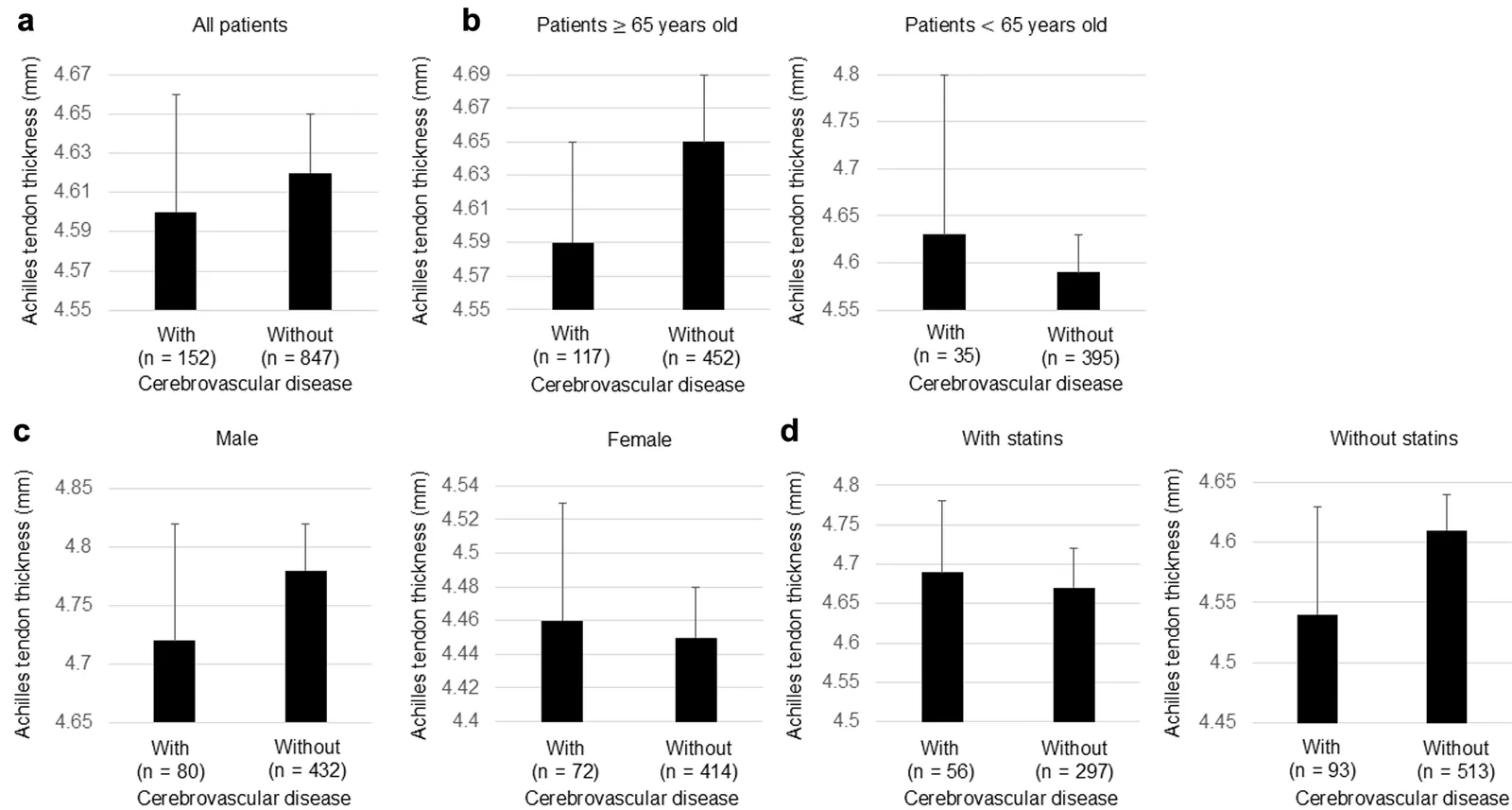
Achilles tendon thickness in patients with and without cerebrovascular disease in all patients (a), in patients ≥ 65 years old and patients < 65 years old (b), in male and female patients (c), and in patients treated with statins and patients untreated with statins (d). Error bars indicate mean ± SE. SE: standard error.

Comparisons of ATT between patients with and without carotid artery stenosis are shown in Figure 3. ATT was significantly greater in patients with carotid artery stenosis than in those without carotid artery stenosis across all patients, patients older than 65 years, and patients treated with statins.

**Figure 3:**
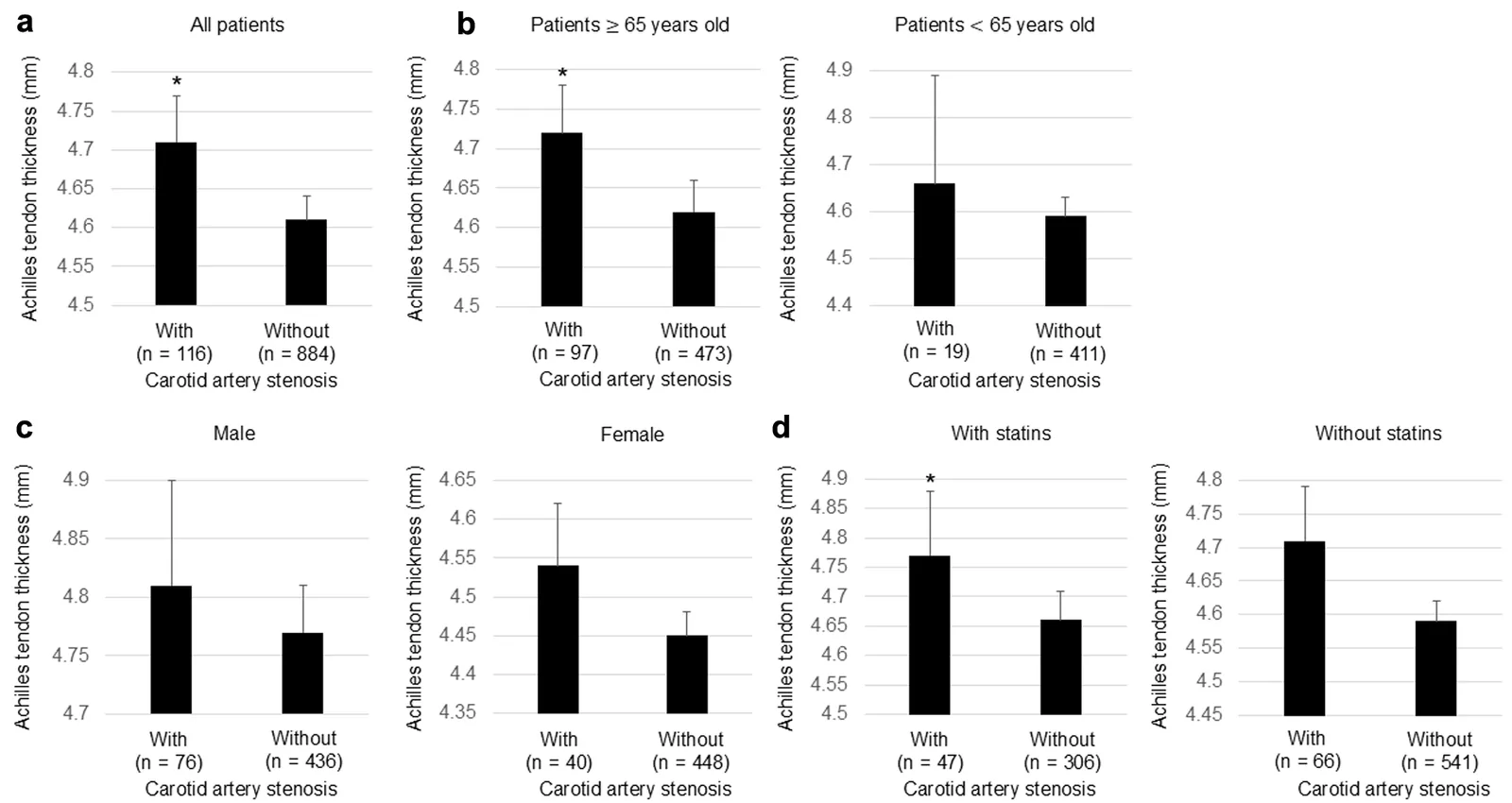
Achilles tendon thickness in patients with and without carotid artery stenosis in all patients (a), in patients ≥ 65 years old and patients < 65 years old (b), in male and female patients (c), and in patients treated with statins and patients untreated with statins (d). Error bars indicate mean ± SE. *P < 0.01 vs. patients without carotid artery stenosis. SE: standard error.

### ATT and diabetes

Comparisons of ATT between patients with and without diabetes are shown in Figure 4. ATT was significantly greater in patients with diabetes than in those without diabetes across all patients, both patients older and younger than 65 years, male and female patients, and patients untreated with statins.

**Figure 4:**
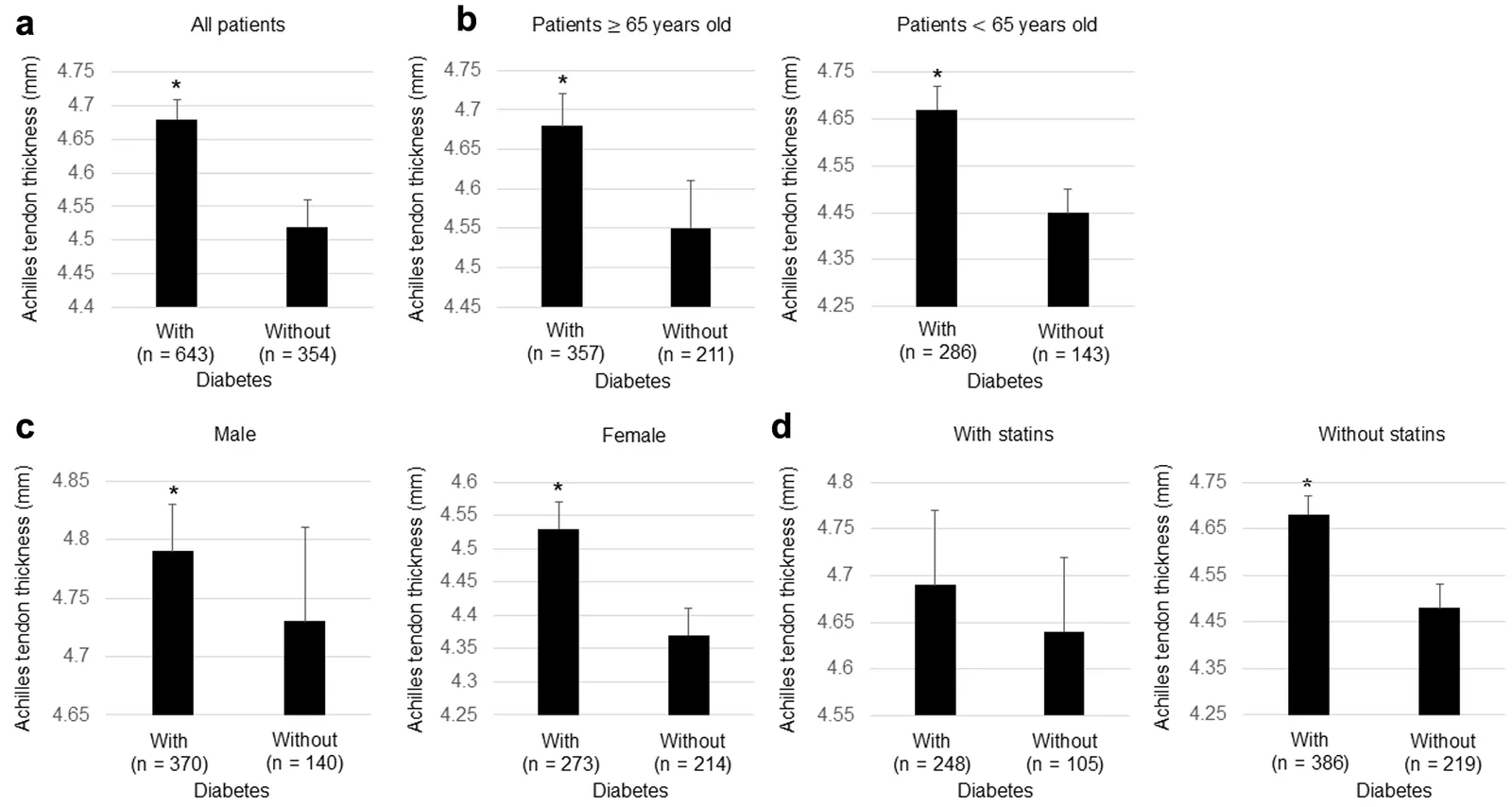
Achilles tendon thickness in patients with and without diabetes in all patients (a), in patients ≥ 65 years old and patients < 65 years old (b), in male and female patients (c), and in patients treated with statins and patients untreated with statins (d). Error bars indicate mean ± SE. *P < 0.01 vs. patients without diabetes. SE: standard error.

### ATT and atherosclerotic diseases in patients with diabetes

Among patients with diabetes, those with CAD had an approximately 37% greater ATT than those without CAD (Fig. 5). Similarly, diabetic patients with carotid artery stenosis had a greater ATT than those without carotid artery stenosis.

**Figure 5:**
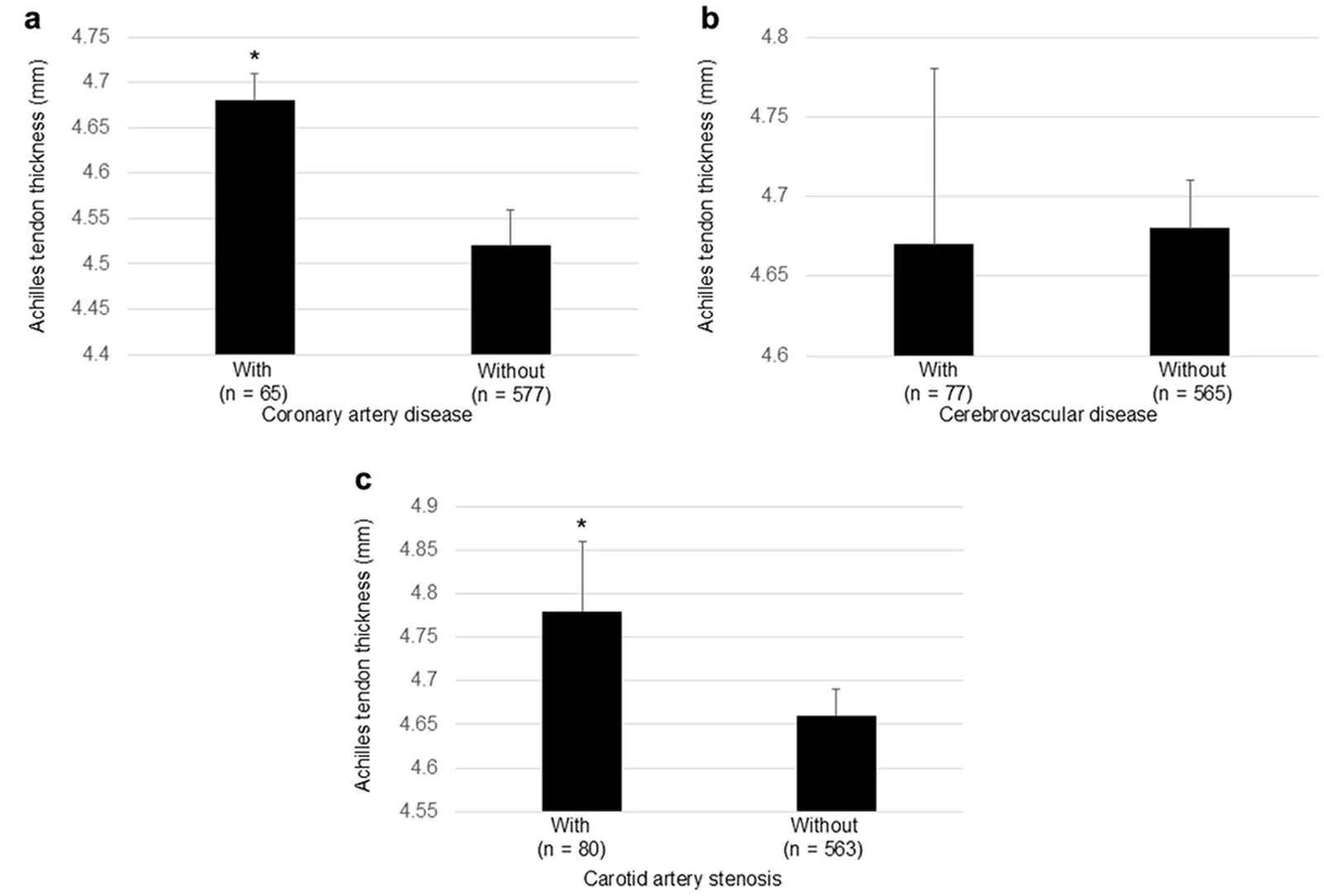
Achilles tendon thickness in patients with and without coronary artery disease (a), cerebrovascular disease (b), and carotid artery stenosis (c) in patients with diabetes. Error bars indicate mean ± SE. *P < 0.01 vs. patients without coronary artery disease (a) and without carotid artery disease (c). SE: standard error.

## Discussion

ATT was originally established as a diagnostic marker for FH, an inherited genetic disorder showing high LDL-C levels due to hepatic LDL receptor defects, tendon xanthomas, and a family history of early-onset ASCVD [1]. FH causes xanthomas, cholesterol deposits most commonly in the Achilles tendon [15]. Patients with FH present with Achilles tendinopathy, such as painful tenosynovitis, before developing tendon xanthomas [16]. Tendon xanthomas consist of cholesterol deposits, inflammatory cells, and a remarkable fibrotic reaction [16].

Extracellular matrix remodeling and inflammation lead to the development of atherosclerosis in patients with hypercholesterolemia-related tendon pathology [17, 18]. The lipid composition of tendon xanthomas, including free cholesterol, cholesterol esters, and phospholipids, suggests systemic lipid infiltration rather than local synthesis [19, 20]. This is supported by observations of active LDL uptake by lesions within xanthomas [21]. The distribution of oxidized LDL parallels that of macrophages in xanthoma [22]. Plasma-derived LDL may be trapped in the tendon matrix and oxidized by macrophages [20]. Most xanthomas originate from macrophages that have taken up oxidized LDL. The role of inflammation in the onset and progression of CVD is well established. Macrophages derived from patients with tendon xanthomas are more likely to form foam cells than macrophages derived from patients without tendon xanthomas [23]. Patients with FH and tendon xanthomas had higher inflammatory cytokine levels than those without. Thus, the pathogenic mechanism underlying the development of tendon xanthomas is similar to that of atherosclerosis.

More recently, ATT has gained attention as a potential predictor of ASCVD, in addition to a diagnostic marker for FH. Ultrasonographic measurement of ATT is now widely used to diagnose FH because of its noninvasive nature, reproducibility, and lack of radiation exposure [8]. A study of patients with heterozygous FH suggested that the ATT assessed by ultrasonography was superior to that assessed by radiography in predicting MACEs [9]. This may partly relate to the complex three-dimensional structure and torsion of the Achilles tendon, which can affect radiographic measurements [24].

Carotid atherosclerotic changes reflect systemic atherosclerosis and serve as a predictive indicator of ASCVD, including cerebrovascular disease and CAD [25–28]. Carotid IMT is a marker of subclinical atherosclerosis. In our study, ATT was positively and significantly correlated with maximal carotid IMT in univariate correlation and the model that included BMI, HDL-C, and LDL-C. In the model that included age as an explanatory variable, the correlations of ATT with BMI and HDL-C remained significant; however, the significant correlation between ATT and maximal carotid IMT was no longer significant.

Obesity and insulin resistance contribute to dyslipidemia by increasing hormone-sensitive lipase activity, enhancing free fatty acid release, and increasing hepatic very-low-density lipoprotein (VLDL) production, while simultaneously reducing lipoprotein lipase (LPL) activity and HDL formation [29, 30]. Briefly, insulin resistance inactivates LPL, the enzyme that catabolizes TG-rich lipoproteins such as VLDL [31]. HDL production is associated with LPL-mediated catabolism of TG-rich lipoproteins [32]. A decrease in LPL activity leads to an increase in VLDL levels and a decrease in HDL levels. In obesity, when inflammatory cells, particularly activated macrophages, infiltrate adipose tissue, a severe inflammatory state is induced [33]. Under such conditions, adipose tissue produces inflammatory cytokines such as tumor necrosis factor-α, which induce atherosclerosis [33]. In our study, body weight and BMI were positively and significantly correlated with ATT. In contrast, HDL-C was inversely correlated with ATT, suggesting that insulin resistance and insulin resistance-induced dyslipidemia are associated with ATT progression. Fujiwara et al reported that maximum ATT had positive correlations with BMI and C-reactive protein [7], supporting our suggestion.

Our study demonstrated that ATT in patients with CAD was significantly higher than in those without CAD in all patients. To elucidate the effects of confounding factors, including age, gender, LDL-C medications, we performed a comparison between patients with CAD and those without CAD, in patients ≥ 65 years old and patients < 65 years old, in male and female patients, and in patients treated with statins and patients untreated with statins. Although no significant difference was observed in some subgroups, no subgroup without CAD exhibited higher ATT values. Similar results were observed in the group comparison regarding carotid artery stenosis. Patients with AT thickening have a higher incidence of multivessel CAD and left main CAD than patients without AT thickening [34]. Furthermore, the presence of AT thickening is associated with vulnerable plaques with macrophage accumulation [34]. In another study of patients with CAD, ATT was independently associated with CAD severity, suggesting that detecting ATT may help identify patients with advanced CAD [35]. A greater ATT may indicate the severity of atherosclerotic lesions, as supported by a greater ATT in patients with carotid artery stenosis than in those without carotid artery stenosis.

While diabetes is an important risk factor for atherosclerosis, ATT in patients with diabetes has been investigated in relation to foot ulcers and peripheral neuropathy [36–39], rather than as a surrogate marker for systemic atherosclerosis. However, one study, albeit with a very small sample size of 43 cases, reported a positive correlation among ATT, CAD, and peripheral artery disease [40]. Our study using 1,000 patients, including 643 with diabetes, showed that ATT levels were higher in patients with diabetes than in those without diabetes in all patients. Significantly higher ATT levels in patients with diabetes were observed regardless of age or gender. In the group of patients treated with statins, there was no significant difference in ATT between those with and without diabetes. However, ATT levels were higher in diabetic patients untreated with statins than in those without non-diabetic patients untreated with statins. Stains may have beneficial effects on ATT. Furthermore, ATT levels were higher in diabetic patients with CAD and carotid artery stenosis than in those without CAD and carotid artery stenosis. In this study, atherosclerotic risk factors, including BMI and low HDL-C levels, were significantly associated with the degree of ATT. These results suggest that the ATT can be used as a surrogate marker for systemic atherosclerosis. There are very few studies examining the correlation between metabolic parameters and ATT levels in diabetic patients. In a study of 289 patients with type 2 diabetes, with and without peripheral neuropathy or foot ulcers, ATT was positively correlated with diabetes duration and HbA1c [41]. To our knowledge, this is the first study to report that factors other than blood glucose control, such as BMI and low HDL-C levels, are associated with ATT in diabetic patients.

This study has several limitations. It is a retrospective observational study, which limits causal inference and directionality. Furthermore, the treatments for diabetes, hypertension, and dyslipidemia, as well as diet and exercise, may have also influenced the results.

### Conclusion

Our findings demonstrate that ATT is significantly associated with cardio-metabolic risk factors, such as BMI and HDL-C. ATT was significantly increased in patients with atherosclerotic diseases, including CAD and carotid artery stenosis. Furthermore, ATT was higher in patients with diabetes compared with non-diabetic individuals, and was further elevated in diabetic patients with CAD or carotid artery stenosis.

## Data Availability

The data supporting the findings of this study are available from the corresponding author upon reasonable request.

## Abbreviations

ASCVD: atherosclerotic cardiovascular diseases
ATT: Achilles tendon thickness
BMI: body mass index
CAD: coronary artery disease
FH: familial hypercholesterolemia
HbA1c: hemoglobin A1c
HDL-C: high-density lipoprotein-cholesterol
IMT: intima-media thickness
JIHS: Japan Institute for Health Security
LDL-C: low-density lipoprotein cholesterol
LPL: lipoprotein lipase
MACEs: major adverse cardiovascular events
SD: standard deviation
TC: total cholesterol
TG: triglyceride
VLDL: very-low-density lipoprotein
